# Radiotherapy and immunology

**DOI:** 10.1084/jem.20232101

**Published:** 2024-05-21

**Authors:** Liangliang Wang, Connor Lynch, Sean P. Pitroda, András Piffkó, Kaiting Yang, Amy K. Huser, Hua Laura Liang, Ralph R. Weichselbaum

**Affiliations:** 1 Department of Radiation and Cellular Oncology, University of Chicago, Chicago, IL, USA; 2 Ludwig Center for Metastasis Research, University of Chicago, Chicago, IL, USA; 3 Department of Neurosurgery, University Medical Center Hamburg-Eppendorf, Hamburg, Germany

## Abstract

The majority of cancer patients receive radiotherapy during the course of treatment, delivered with curative intent for local tumor control or as part of a multimodality regimen aimed at eliminating distant metastasis. A major focus of research has been DNA damage; however, in the past two decades, emphasis has shifted to the important role the immune system plays in radiotherapy-induced anti-tumor effects. Radiotherapy reprograms the tumor microenvironment, triggering DNA and RNA sensing cascades that activate innate immunity and ultimately enhance adaptive immunity. In opposition, radiotherapy also induces suppression of anti-tumor immunity, including recruitment of regulatory T cells, myeloid-derived suppressor cells, and suppressive macrophages. The balance of pro- and anti-tumor immunity is regulated in part by radiotherapy-induced chemokines and cytokines. Microbiota can also influence radiotherapy outcomes and is under clinical investigation. Blockade of the PD-1/PD-L1 axis and CTLA-4 has been extensively investigated in combination with radiotherapy; we include a review of clinical trials involving inhibition of these immune checkpoints and radiotherapy.

## Teaser

The majority of cancer patients receive radiotherapy. Wang et al. review preclinical and clinical data related to the importance of immune responses to radiotherapy-induced anti-tumor effects, as well as blockade of immune checkpoints PD-1/PD-L1 and/or CTLA-4 in combination with radiotherapy.

## Introduction

Radiotherapy is a form of cancer treatment delivered to ∼50% of all cancer patients that relies on the spatial delivery of ionizing radiation (IR) to kill tumor cells while minimizing the effects on normal tissues. Radiotherapy is employed with the intention of curing or for relief of symptoms, termed palliation. The first description of irradiation as a treatment emerged from Emil Grubbé in Chicago in 1896, and it was administered for postoperative breast cancer recurrence (Hodges, 1964). Early research and clinical efforts were limited to surface x-ray irradiations; technological developments during World War I produced sources capable of 200 kV and deeper penetration (Del Regato, 1996). Advances during and following World War II led to post-war use of cobalt 60 sources, which emit γ radiation, as well as betatrons and linear accelerators (LINACs), which both use high-energy x-rays (Del Regato, 1996; Fowler, 2006; Hellman, 1996). γ Radiation produces rays of the shortest wavelength and features energetic photons; high-energy x-rays have a wider wavelength and emit electrons (Hall and Giaccia, 2018). Radiotherapy is delivered either by external beam with x-rays, γ rays, or photons, or internally, by implantation of radioactive sources into or near the tumor (brachytherapy) or through the delivery of untargeted (^131^I) or targeted (^177^Lu-PSMA) radioisotopes. These latter internally administered charged particle therapies rely on the short half-life and rapid spatial falloff in a dose associated with mixed β and γ emitters such as ^192^Ir, which is commonly used in brachytherapy, and the radioisotopes ^131^I and ^177^Lu. There is significant interest in the use of protons to deliver radiotherapy due to improved dose distributions; however, except for use in pediatrics, the clinical benefit of protons compared with standard LINAC-delivered radiotherapy remains controversial.

Radiotherapy is most often delivered with the goal of improving local control, which, in many instances, is curative or part of a curative multimodality regimen. For example, in many cases of localized breast cancer, the tumor is excised and radiotherapy is delivered to preserve cosmesis, along with hormonal therapies or chemotherapy aimed at curing microscopic distant metastasis. Other examples include the use of cisplatin with radiotherapy in head and neck cancer to improve local cures, as well as cisplatin in cervix cancer with external radiotherapy and (internal) brachytherapy. Most recently, the treatment of a few metastases (termed “oligometastasis”) has brought radiotherapy into the arena of potentially curing subsets of patients with metastatic disease. The combinations of radiotherapy and immunotherapy are an area of intense research interest and will be discussed in detail below.

The key cytotoxic mechanism of action of radiotherapy involves induction of various forms of DNA damage, including double-strand breaks, which can be repaired by several highly conserved repair pathways, and repair is initiated almost immediately upon sensing of DNA damage (McBride et al., 2004). Also, microenvironmental factors such as tumor hypoxia have been implicated in radiotherapy failure, although like most cytotoxic therapies, tumor volume is the most likely determinant of cure by radiotherapy. Certain histological subtypes such as glioblastoma and pancreatic cancer seem unusually refractory to radiotherapy or radiochemotherapy treatment. The effects of IR on both tumors and normal tissues are determined by the overall dose, the daily dose (fraction size), and the overall time of delivery. The most common overall dose is 50–60 Gy in 1.8–2 Gy daily fractions delivered externally by a LINAC. Alternative delivery strategies, such as stereotactic body radiotherapy (SBRT, using a few large doses in a short period of time), more than one treatment per day (hyper-fractionation), or a slightly larger dose than standard (2–2.5 Gy/day) over a shorter period of time (accelerated fractionation or hypo-fractionation) are employed to improve treatments by killing more tumor cells and decreasing tumor repopulation between doses (Hall and Giaccia, 2018). Technological advances such as intensity-modulated radiotherapy, proton therapy, and improved imaging techniques integrated into therapy machines are employed to improve the precision of dose delivery to tumors. The immune contexture of the tumor may be a determinant of radiocurability, and recent studies have been aimed at exploiting the immune system with radiotherapy to improve the therapeutic index.

## Preclinical studies of radiation and anti-tumor immunity

**Figure 1.**
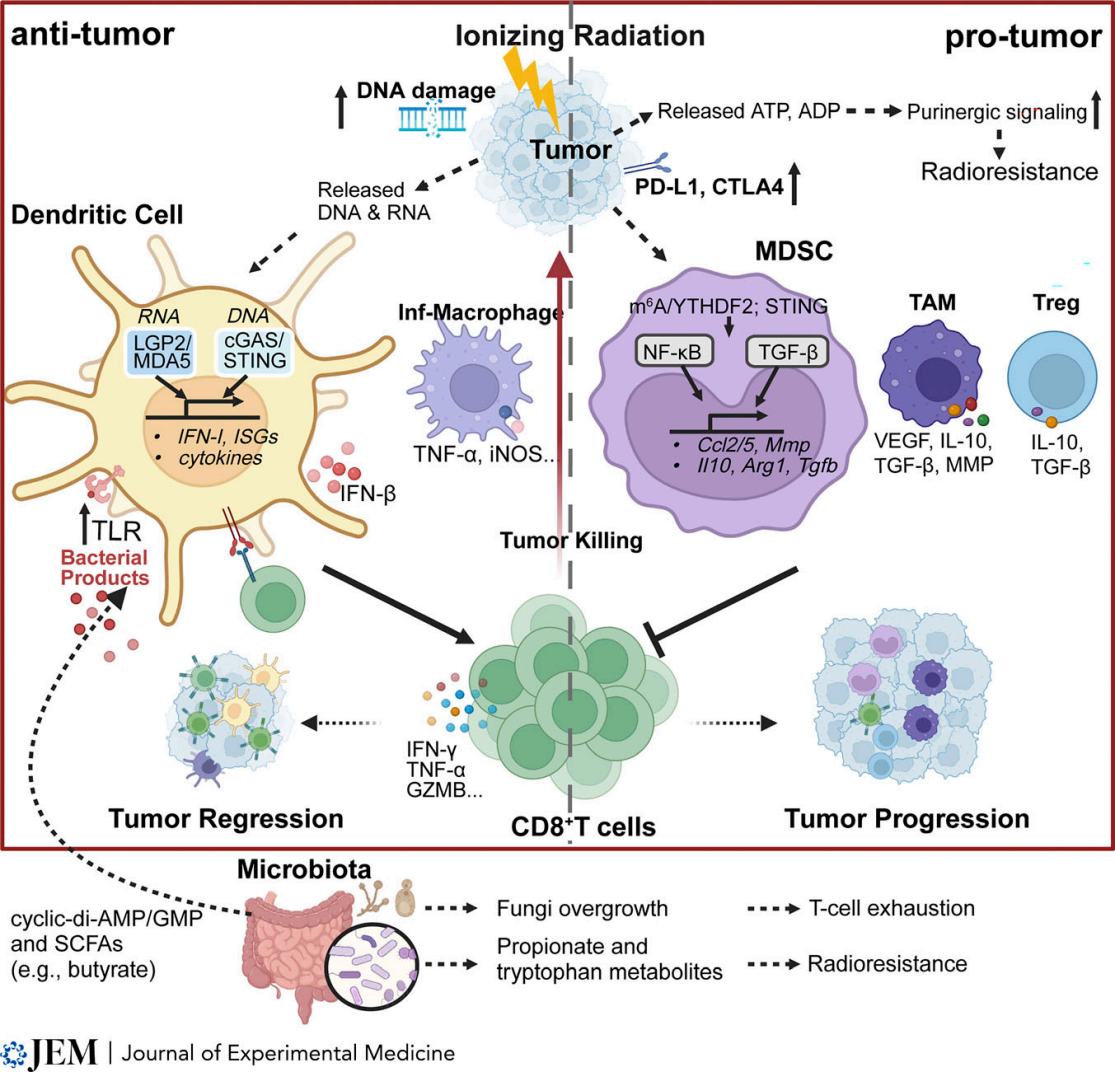
**IR reprograms the TME, initiating both pro-tumor and anti-tumor effects.** Host immune status and other factors, such as microbiome populations, can affect tumor immunity. Left: Anti-tumor effects of IR. DCs are activated by RNA and DNA sensing pathways, which results in production of type I IFN, IFN-stimulated genes (ISGs), cytokines, and chemokines. DCs and inflammatory macrophage activate T cells to produce more IFN-γ, TNF-α, and granzyme B (GZMB), and kill tumor cells. Right: Pro-tumor effects of IR. IR induces PD-L1 and CTLA-4 expression in tumors. IR also induces infiltration of MDSCs, TAMs, and Tregs, which collectively inhibit T cell cytotoxic function and promote tumor growth. IR also induces overexpression of purinergic signaling to introduce radioresistance. Bottom: Outside of the tumor, products of certain bacteria strains activate or inhibit DC functions via TLRs. Fungus overgrowth or certain metabolites result in T cell exhaustion or radioresistance, respectively. MMP, matrix metalloproteinase; SCFA, short chain fatty acid. Figure created with BioRender.

Radiation, as an acute insult, results in acute inflammation and, as depicted in Fig. 1, a complex response in the tumor microenvironment (TME). The local inflammation and the overproduction of chemokines lead to increases in immune cells and T-lymphocyte infiltration. By inducing immunogenic cell death, IR generates neoantigens in tumor cells (Reits et al., 2006). IR induces expression of MHC class I molecules on tumor cells, which can result in either increases in existing tumor neoantigen expression or neoantigens from DNA damage–induced mutations (McLaughlin et al., 2020). Recently, Lhuillier et al. reported identifying neoantigens to tumor-specific T cells from somatic nonsynonymous mutations that were overexpressed after IR. Vaccination using neopeptides enhances efficacy of IR in a murine model of triple-negative breast cancer (Lhuillier et al., 2021).

Professional antigen-presenting cells most likely are required for T cell priming at higher potency. Radiation leads to enhanced dendritic cell (DC) maturation and antigen presentation capacity (Burnette et al., 2011; Lugade et al., 2005), followed by T cell priming (Lee et al., 2009). CD8^+^ T cells are required for the optimal anti-tumor effects of IR. Data indicate that a state of equilibrium between cytotoxic CD8^+^ T cell function and tumor cell proliferation can be disrupted through administration of interferon (IFN) γ to tumors, leading to tumor rejection (Liang et al., 2013a, 2013b). IFNβ induction and signaling by IR is critical for tumor response (Ranoa et al., 2016). DCs deficient in IFNβ signaling can contribute to abrogation of IR anti-tumor effects (Chen et al., 2019).

### CD8^+^ T cells are required for IR-induced anti-tumor efficacy

T cells, especially CD8^+^ T cells, activated (primed) by antigen-presenting cells (such as DCs) are well documented to be required for IR-induced anti-tumor immunity. IR not only increases the infiltration of T cells but also enhances the cytotoxicity of T cells by augmenting the production of tumor necrosis factor (TNF) α, IFNγ, and granzyme B (Hay and Slansky, 2022; Kohno et al., 2020; Park et al., 2014). In almost all murine tumor models, CD8^+^ T cells are essential since depleting CD8^+^ T cells markedly reduces the anti-tumor effects of IR (Burnette et al., 2011; Deng et al., 2014a; Lee et al., 2009; Liang et al., 2013b; Weichselbaum et al., 2017). Existing data do not indicate an important role for CD4^+^ T cells in response to IR in most murine tumor models, with the exception of a few studies testing combinations: IR + all-trans retinoic acid, where CD4^+^ T cells were required for the manifestation of full anti-tumor effects (Rao et al., 2021); IR + vaccination of CD4^+^ antigen, where CD4^+^ T cells are posited to be important for sustained CD8^+^ T cell activation (Lhuillier et al., 2021); and combination IR + monophosphoryl lipid A treatment, which generates a systemic anti-tumor immune response in a Th1-CD4^+^ T cell–dependent manner in murine melanoma and prostate cancer models (Jagodinsky et al., 2022). Although IR alone activates T cells, they are often quickly exhausted or limited by TME-expressing immune checkpoints induced by IR, such as PD-L1 and CTLA-4 (Gough and Crittenden, 2022).

## IR-induced suppression and negative regulators of anti-tumor immunity

Although IR has been shown to induce cancer cell death and tumor-specific adaptive immune responses, it has been hypothesized that IR-induced suppression (both tumor cell–intrinsic resistance and host cell–extrinsic effects) may account for some treatment failures. Ewing (1917) first observed early lymphocyte loss following radium treatment for cervical cancer (Schaue, 2017), as well as the accumulation of lymphocytes, leukocytes, plasma cells, and polyblasts at later time points, and argued that this immune activity was key to tumor cell killing and normal tissue healing. While lymphopenia continues to present a considerable challenge in radiotherapy (Kleinberg et al., 2019), the response of lymphoid and myeloid subpopulations to IR may hold the key to improving radiotherapy (Pham et al., 2023).

### IR-induced regulatory T cells (Tregs)

Tregs are a unique subpopulation of CD4^+^ T cells that are characterized by expression of the forkhead box P3 transcription factor and high levels of CD25. Tregs exert “unproductive” immunosuppression leading to unwanted immunosuppressive effects or even promoting tumor progression or metastasis (Wolf et al., 2015). Current findings on the effect of IR on Tregs in the TME indicate a complex picture. It has been reported that IR can increase the recruitment of Tregs into the TME in multiple cancers, which may contribute to radioresistance. IR significantly increases tumor-infiltrating Tregs in multiple murine tumor models including melanoma, B cell lymphoma, and prostate cancer (Dutt et al., 2018; Kachikwu et al., 2011; Wolf et al., 2015). In several clinical trials, radiotherapy (including chemo-radiotherapy or SBRT) increases levels of circulating Tregs in the peripheral blood mononuclear cells of patients undergoing treatment for head and neck cancer (Schuler et al., 2013), cervical cancer (Battaglia et al., 2010; Qinfeng et al., 2013), and glioma (Fadul et al., 2011) compared with patients not receiving radiotherapy. Although there is a general consensus that Tregs are immunosuppressive and may contribute to treatment failure, the ratio of Tregs and CD8^+^ effector T cells as well as the timing of changes require further elucidation to comprehensively clarify the relevance of Tregs to the anti-tumor response.

### IR-induced myeloid-derived suppressor cells (MDSCs)

In the context of IR, the expansion of MDSCs and Tregs has been reported to suppress CD8^+^ T cell response in the TME, acting as extrinsic radioresistance (Pointer et al., 2022). Two recent reviews have summarized the role of MDSCs following IR in preclinical models (Jiménez-Cortegana et al., 2022; Wang et al., 2023a). MDSCs develop from common myeloid progenitor cells in the bone marrow, expand in the peripheral blood, and then are attracted to the tumor sites via a variety of cytokines and chemokines (Veglia et al., 2021). MDSCs are a heterogeneous, immature population of myeloid cells. Known for their “plasticity” (Schouppe et al., 2012), MDSCs are associated with tumor-associated macrophages (TAMs) and tumor-associated neutrophils (TANs) in the context of cancer: polymorphonuclear-MDSCs seem to develop into N2 TANs while monocytic (M)-MDSCs are proposed to develop into TAMs (Li et al., 2021a).

Local IR can significantly increase circulating MDSCs in humans (Ghosh et al., 2023) and in tumor-infiltrating MDSCs in various murine models including breast cancer, colon cancer, glioma, hepatocellular carcinoma (HCC), lung cancer melanoma, prostate cancer, and pancreatic cancer. The induction of MDSCs by IR in syngeneic mouse models of cancer depends on different signaling pathways/mechanisms. Xu and colleagues highlighted the importance of CSF1/CSF1R signaling in the recruitment of CD11b^+^Ly6G^+^ MDSCs in irradiated tumors (Xu et al., 2013). The Warburg effect–induced lactate secretion has been shown to trigger IR-mediated MDSC expansion in an HIF-1α/STAT3-dependent manner in murine pancreatic cancer (Yang et al., 2020). Previous investigations demonstrated that activation of the cyclic GMP-AMP synthase/stimulator of IFN genes (cGAS/STING) pathway in MDSCs was essential for the attraction of monocyte MDSCs in a CCR2-dependent manner in a colon cancer model (Liang et al., 2017). YTHDF2–NF-κB signaling was recently implicated as a mechanism of IR-induced MDSC tumor infiltration (Wang et al., 2023a). In cancer patients, the induction of MDSCs by radiotherapy has been observed in the spleen, lung, lymph nodes, and peripheral blood (Xu et al., 2013), and thereby contributes to suboptimal anti-tumor effects.

### IR-induced TAMs

TAMs are among the most represented cells of the immune system in the TME and can be derived from tissue-resident progenitors or circulating monocytes/M-MDSCs (Cassetta et al., 2019; Cassetta and Pollard, 2023; Nalio Ramos et al., 2022). TAMs contribute to multiple crucial steps of tumorigenesis (Kloosterman and Akkari, 2023) including, but not limited to, angiogenesis (Jetten et al., 2014; Lin et al., 2006), suppression of adaptive immune responses (Doedens et al., 2010; Fan et al., 2021), and establishment of the premetastatic niche (Casanova-Acebes et al., 2021; Gil-Bernabé et al., 2012). Historically, macrophages were dichotomized into M1 (anti-tumorigenic) and M2 (pro-tumorigenic) macrophages, and it was suggested that IR can induce an M1-like phenotype with increased inducible nitric oxide synthase expression (Klug et al., 2013; Rao et al., 2021; Teresa Pinto et al., 2016), and that M2-like macrophages exhibit higher radioresistance (Leblond et al., 2017). However, since the advent of small-cell RNA sequencing studies, the concept of M1/M2 polarization is being increasingly abandoned (Ginhoux et al., 2016; Nahrendorf and Swirski, 2016), and a diverse spectrum of multiple TAM phenotypes is now being recognized (for recent consensus review, see Ma et al., 2022). It has furthermore been shown that IR can accelerate the differentiation of infiltrating monocytes toward TAMs expressing immunosuppressive gene signatures (Wang et al., 2023a). More research is needed to understand the differential effects of IR on specific TAM subsets and how this could be leveraged therapeutically.

## The crucial signaling pathways activated by IR or IR+immune checkpoint blockade (ICB)

### DNA damage sensing

IR, like other DNA-damaging agents, results in genomic instability and release of double-stranded DNA (dsDNA) or RNA into the cytosol of dying or stressed cells. These nucleic acids are taken up by tumor-associated DCs and trigger a series of events (discussed in detail in a later section) to activate antigen cross-presentation of DCs. The activation of DCs by increased levels of IFNβ and an abundance of neoantigens leads to enhanced local and systemic adaptive responses (Weichselbaum et al., 2017). It has been reported that TREX1, a DNA exonuclease, is activated by doses higher than 12–18 Gy, which degrades DNA and impairs the IFN response (Vanpouille-Box et al., 2017).

Pattern recognition receptors (PRRs) are important components of the innate immune system (Hoffmann and Akira, 2013). Nucleic acid sensor PRRs can be divided into different categories: (i) sensors that detect nucleic acids in endosomes, such as Toll-like receptor (TLR) family members; (ii) sensors of nucleic acids in cytosol, such as retinoic acid–inducible gene-I (RIG-I)–like receptors (RLRs) and cGAS; and (iii) extracellular RNA and DNA sensing. IR can induce cellular immune responses in cancer cell lines by triggering their RNA virus sensor pathway (Khodarev, 2019). IR stimulates the binding of RIG-I to small nuclear RNAs U1 and U2 and results in IFNβ production. Deletion of RIG-I in cancer cells renders them resistant to IR and chemotherapy (Ranoa et al., 2016). IR is reported to induce long terminal repeats (LTRs), which are key ligands for the RNA virus sensor RIG-I (Du et al., 2023). The activated mTOR–LTR–RIG-I axis induces cellular immune response by dramatically increasing inflammatory cytokine and chemokine production, potentially enhancing DC and macrophage infiltration after irradiation. Preclinical findings indicate that host RNA sensing activation is required for the anti-tumor action of IR; RLR LGP2-deficient mice lose the ability to respond to IR (Zheng et al., 2020). However, expression of the RLR LGP2 contributes to radioresistance (Widau et al., 2014).

### cGAS/STING/IFN-I

Radiation induces DNA damage, which leads to dsDNA breaks, and these can be sensed by cell-intrinsic or -extrinsic mechanisms. DNA sensors AIM2, in the nucleus (Hu et al., 2016), and cGAS, in the cytosol (Cai et al., 2014), recognize the IR-induced dsDNA, and this triggers a cascade of downstream events to mount an inflammatory reaction and innate immunity (Fig. 1). Cytosolic DNA activates cGAS to synthesize 2′3′-cGAMP from ATP and GTP. As a high-affinity ligand for STING, 2′3′-cGAMP binds and activates STING and the STING/TBK1/IRF3 axis, which induces the production of type I IFNs and other cytokines. IR can induce STING activation, presumably by inducing DNA damage and double-stranded breaks. Meiotic recombination 11 homolog A serves as a cytosolic sensor for dsDNA and is required for STING/IRF3 activation (Kondo et al., 2013), via liberating cGAS from nucleosome sequestration (Cho et al., 2024). DNA-dependent protein kinase (DNA-PK) phosphorylates and suppresses cGAS to diminish antiviral innate response (Sun et al., 2020). By contrast, DNA-PK may lead to cGAS/STING-independent IFN production to boost innate immune response (Burleigh et al., 2020), depending on biological context (Berger et al., 2022).

STING, especially STING in DCs, is required for innate immunity activation and IR-induced anti-tumor effects (Deng et al., 2014b; Wu and Murphy, 2022). Potent bacterial-derived STING agonist Bis-(3′-5′)-c-di-AMP delivered by zinc nanoparticle, used alone or in combination with IR, exhibited superior anti-tumor activity (Yang et al., 2022). The anti-tumor action requires STING activation in endothelial cells, which eventually destroys tumor vasculature. STING activation in TME also reprogrammed macrophages to present tumor antigens. Recently, natural killer (NK) cells were shown to be critical for tumor inhibition induced by a STING agonist. However, STING activation in different stages of tumorigenesis, in different cell types, at different times, and different IR doses may play distinct and sometimes contrasting roles. For example, cancer cell–intrinsic STING activation may cause chronic inflammation and lead to tumor progression (Kumar et al., 2023). STING activation in phagocytic MDSCs results in production of both proinflammatory cytokines and anti-inflammatory IL-10 (Ahn et al., 2017). High doses (12–18 Gy) of IR changes the expression of DNA exonuclease TREX1, which in turn inhibits STING activity (Vanpouille-Box et al., 2017). cGAS/STING pathway activation leads to production of inflammatory cytokines and chemokines, including CCL-2, -3, -5, -7, and -12, which increases infiltration of M-MDSCs into the TME. The rupture of micronuclei generated by chromosomal instability triggers activation of the cGAS/STING pathway and promotes metastasis (Bakhoum et al., 2018). The complexity of the cGAS/STING cascade likely underlies the negative results in clinical trials using STING agonists alone or in combination with other therapies to date, although investigations continue (Motedayen Aval et al., 2020).

### The role of the purinergic signaling pathway in radioresistance in the context of anti-tumor immunity

The purinergic signaling pathway is formed by nucleotides/nucleosides (main cellular messengers including ATP, ADP, adenosine [ADO], uridine triphosphate, and uridine diphosphate) with their corresponding membrane receptors (Huang et al., 2021). Following release into extracellular space, ATP can be dephosphorylated/hydrolyzed by ectonucleotidases including ectonucleoside triphosphate diphosphohydrolases (like CD39/NTPDase 1), ecto-5′-nucleotidase (CD73/NT5E), ectonucleotide pyrophosphatase/phosphodiesterase, and alkaline phosphatase (Yegutkin, 2014). These various nucleotides activate their corresponding receptors, such as ATP to (ligand-activated ion channels) P2YR receptors (belonging to superfamily of G-protein coupled receptor), and downstream kinase signaling (Stagg and Smyth, 2010). Purinergic signaling coordinates cancer-cell proliferation, differentiation, migration, and different types of apoptosis, as well as immune responses and inflammation responses (Eltzschig et al., 2012; Huang et al., 2021), performing fundamental dual roles in the TME. Here, we will mainly discuss suppressive effects in the context of IR and ICB.

Accumulating evidence supports the idea that the purinergic signaling pathway, involving ATP, ADP, CD39, and CD73, plays an important role in the resistance of cancer cells to IR. Radiation induces the release of ATP into the TME. The induction of ATP by IR is independent of the dose, since a low dose of 0.25 Gy up to 8 Gy can accelerate ATP release in human and murine cancers (Kojima et al., 2004; Zanoni et al., 2022). In addition, the stimulation of ATP may be rapid or relatively long-lasting (5 min after IR to 21 days after IR) (Kojima et al., 1997; Zanoni et al., 2022). We speculate that low-dose, not high-dose, irradiation induces release of ATP.

ATP is reported to mediate radioresistance by promoting proliferation, migration, and epithelial-to-mesenchymal transition (EMT) of cancer cells in a variety of cancers. IR-induced ATP can bind to receptors (mainly P2X7R and P2Y2R) in cancer cells to form an autocrine–paracrine signaling loop. ATP plays a role in radioresistance by promoting IR-induced DNA damage repair. IR-induced ATP activates P2Y6 and/or P2Y12, resulting in formation of foci consisting of γH2AX, 53BP1, and ataxia telangiectasia mutated (ATM), which mediates repair of DNA damage in an ERK1/2-dependent manner (Ide et al., 2014; Nishimaki et al., 2012).

IR-induced ATP release contributes to the differentiation of naive CD4^+^ T cells into Tregs by stimulating the adenosine A2B receptor (Kojima et al., 2017; Nakatsukasa et al., 2011). ADO contributes to the differentiation from monocyte into TAM, and ADO abolishes the phagocytic activity of macrophages, which amplifies the immunosuppressive function (Belikoff et al., 2011). Release of ATP or ADO in the context of IR could be one mechanism of radioresistance and a feasible target for drug development for cancer treatment.

### The immune checkpoint pathways

The immune checkpoint pathways are a major mechanism of immune resistance during cancers. Among these, the coinhibitory molecule programmed cell death protein 1 (PD-1) and its ligand programmed cell death ligand 1 (PD-L1) play a central role in inhibiting anticancer T cell immunity (Li et al., 2018). The significance of the PD-L1/PD-1 axis to cancer treatment is that high expression of PD-L1 has been observed in multiple types of human cancers including non-small cell lung cancer (NSCLC), melanoma, renal cell carcinoma, prostate cancer, and gastric cancer (Cha et al., 2019). In addition to tumor cells, it has been reported that tumor-infiltrating immune cells also express PD-L1 and contribute to pro-tumor activities (Lin et al., 2018). Recent studies focused on exosomal PD-L1 in the extracellular vesicles and soluble PD-L1 in the blood indicate similar immunosuppressive function (Chen et al., 2018; Finkelmeier et al., 2016; Poggio et al., 2019; Rossille et al., 2014).

Increasing evidence has demonstrated that radiation induces an increase in expression of PD-L1 on tumor cells in multiple cancers including colon, head and neck, squamous cell carcinoma, breast cancer, nasopharyngeal carcinoma, prostate cancer, and glioma (Chen et al., 2023b; Ding et al., 2020; Dovedi et al., 2014; Oweida et al., 2017; Wu et al., 2016). Radiation induces PD-L1 expression in tumor cells via various pathways like DNA damage/ATM/STAT3/IRF3 axis, IFN-γ/JAK/STAT/PD-L1 pathway, cGAS-STING signaling, epidermal growth factor receptor (EGFR)/PI3K-AKT cascade, and the HIF-1α signaling (Ding et al., 2021; Wang et al., 2022). Of note, other signaling pathways may be involved in the regulation of PD-L1 expression in tumors.

Much investigation has focused on PD-L1 expression in MDSCs: single high-dose IR (15 or 20 Gy) can significantly elevate the PD-L1 expression (Deng et al., 2014a). Ablative hypofractionated IR (40 Gy in four fractions) also increased PD-L1 expression in a murine lung cancer model (Chen et al., 2021). However, it remains unclear which signaling pathways are involved in PD-L1 expression in MDSCs or other tumor-infiltrating immune cells. Radiotherapy-induced PD-L1 expression and the consequent immunosuppression provides the rationale for combinations involving anti-PD-L1 immunotherapy for cancer treatment.

Cytotoxic T-lymphocyte-associated protein 4 (CTLA-4) is another inhibitory receptor, and the interaction between CTLA-4 and its ligands potentially inhibits T cell activation (Fife and Bluestone, 2008). CTLA-4 engagement blocks the activation of transcription factors such as NF-κB, nuclear factor of activated T cell, and AP-1 in activated T cells, limits IL-2 production, and thereby dampens immune responses (Seidel et al., 2018). It has been reported that radiotherapy enhances systemic responses to anti-CTLA-4 antibodies in preclinical and clinical studies (Demaria et al., 2016; Formenti et al., 2018). T cell immunoglobulin mucin-3 (TIM-3) is a negative regulator of lymphocyte function and acts as a marker for T cell exhaustion (Zhu et al., 2011). Targeting TIM-3 in combination with radiotherapy resulted in improved murine cancer inhibition and animal survival (Kim et al., 2017). A recent study reported that blocking the T cell immunoreceptors with the immunoglobulin and immunoreceptor tyrosine-based inhibitory motif domain can improve the response to radiotherapy in murine cancer models (Zhao et al., 2023). Targeting other immune checkpoints (like LAG-3, BTLA) combined with radiotherapy in cancer treatment is an area of interest with potential implications for immunotherapy and needs more (pre-)clinical investigation in the future.

### NF-κB signaling in both tumor cells and host cells

IR can activate NF-κB signaling in host immune cells, playing a fundamental role in influencing immune response. A recent study reported that IR or irradiated tumor cells activated NF-κB signaling in MDSCs, improved the differentiation, migration, and suppressive function of tumor-infiltrating MDSCs, and thereby impaired the CD8^+^ T cell response (Wang et al., 2023a). Therefore, understanding the dynamic regulation of NF-κB signaling in both tumor cells and immune cells in response to IR is essential for developing therapeutic strategies to enhance the effectiveness of radiotherapy.

### IR-induced TGF-β signaling

Although context-dependent under many circumstances, TGF-β is one of the most potent suppressors of immune activity against cancer cells. TGF-β signaling has been implicated in promoting radioresistance, and IR induces TGF-β within the TME. Tumor cells, monocytes, macrophages, and platelets have all been reported to be sources of TGF-β following IR (Farhood et al., 2020; Xia et al., 2014; Yarnold and Brotons, 2010). The elevated TGF-β levels decrease the sensitivity of cancer cells to IR, likely via the phosphorylation of H2AX, ATM, and p53 during DNA damage responses of cancer cells and via HIF-1α/vascular endothelial growth factor (VEGF) activation for angiogenesis (Farhood et al., 2020). TGF-β signaling can induce EMT in both suppressor of mothers against decapentaplegic (SMAD)–dependent and non-SMAD-dependent manners following IR (Wang et al., 2021; Yadav and Shankar, 2019).

TGF-β also plays a crucial role in regulating the immune system during radiotherapy (Vanpouille-Box et al., 2015). Following IR, TGF-β can upregulate the expression of PD-1 and CTLA-4, which promotes apoptosis in cytotoxic CD8^+^ T lymphocytes (CTLs) and DCs (Bai et al., 2019; Rekik et al., 2015). TGF-β can increase the proliferation of Tregs within the TME (Santarpia et al., 2015). TGF-β signaling is required for MDSC migration and suppressive function after IR (Wang et al., 2022). TGF-β signaling triggers the polarization of M2-type macrophages and the differentiation of suppressive monocytes, thereby promoting tumor progression (Novitskiy et al., 2012; Pang et al., 2013). These changes lead to suppression of CTLs and NK cells and consequent reduction in cancer-cell killing (Farhood et al., 2020). Overall, TGF-β acts as a radiation protection agent, leading to heightened interest in TGF-β as a therapeutic target.

### The role of *N*^6^-methyladenosine (m^6^A) methylation in radioresistance

Among RNA modifications in eukaryotes, m^6^A is the most common. The m^6^A “writers,” “erasers,” and “readers” work jointly to form a dynamic m^6^A modification process and affect the fate of modified RNA, including its stability, transport, and processing (Frye et al., 2018; He and He, 2021; Shi et al., 2019). Increasing evidence suggests m^6^A modification is implicated in tumor progression, development, and tumor immunity (Liu et al., 2019), and is closely associated with the efficacy of chemotherapy, radiotherapy, and immunotherapy (Deng et al., 2023). m^6^A methylation can be induced by radiotherapy. Shao and colleagues reported that IR (4 Gy) significantly increased the m^6^A levels in several colorectal cancer cells, probably due to the reduced m^6^A eraser ALKBH5 after IR (Shao et al., 2023). A recent report noted that IR increases m^6^A reader YTHDF2 in MDSCs both in vitro (4 Gy) and in vivo (one dose of 20 Gy) (Wang et al., 2022, 2023a). These data suggest that IR-mediated alterations in expression of m^6^A proteins may impact anti-tumor efficacy.

Several studies have reported that RNA m^6^A modification was involved in DNA damage repair induced by IR (Xiang et al., 2017; Yang et al., 2021; Zhang et al., 2020). m^6^A modifications contribute to radioresistance across various cancer types through different mechanisms (Wang et al., 2023b). Most recently, findings in glioblastoma indicate that the m^6^A demethylase ALKBH5 is overexpressed in stem cells, promotes the expression of homologous repair–associated genes, including *Rad51*, *XRCC2*, *BRCA2*, and *EXO1*, and thereby induces radioresistance (Kowalski-Chauvel et al., 2020). In hypopharyngeal squamous cell carcinoma, methyltransferase METTL3 confers radioresistance by reducing tumor cell death via upregulating circCUX1 in an m^6^A manner and the downstream caspase (Wu et al., 2021). In nasopharyngeal carcinoma, m^6^A reader YTHDC2 increased the translation of *IGF1R*, consequently activating IGF1R-AKT signaling, inhibiting tumor cell apoptosis, stimulating protein synthesis, and promoting radioresistance (He et al., 2020). YTHDC1 was also found to promote the transcription of *SREBF1* and the downstream ferroptosis genes, and thereby induce radioresistance.

We assert that modulation of the m^6^A writer/reader axis may be important in the context of IR, and it may be a feasible biomarker for assessment of IR dose using available patient sequencing data and real clinical patient samples for validation.

## Interaction of the microbiome, radiotherapy, and the immune system

The commensal microbiota can alter the effectiveness of various cancer therapies; recent data suggest that the interplay between the microbiome and IR shapes anti-tumor immune responses and may influence treatment outcomes and side effects (Daillère et al., 2016; Geller et al., 2017; Gopalakrishnan and Jenq, 2018; Iida et al., 2013; Matson et al., 2018; Routy et al., 2018a, 2018b; Shiao et al., 2021; Sivan et al., 2015; Viaud et al., 2013).

Clinical studies have identified microbials such as *Bifidobacterium* and *Akkermansia muciniphila* that correlate with heightened immune activation, leading to improved outcomes in patients being treated with immunotherapies (Derosa et al., 2022; Li et al., 2022; Matson et al., 2018; Shi et al., 2020). Analysis by Li et al. (2022) of the fecal samples from 24 patients receiving radiotherapy for HCC revealed that non-responding patients demonstrated a different composition and abundance of bacterial communities compared with responders and healthy controls (Li et al., 2022). This disruption of the gut microbiota was found to deleteriously affect anti-tumor immune responses. Analysis using liquid chromatography high-resolution mass spectrometry revealed that levels of the bacterium-derived cyclic (c)-di-AMP, which promotes the secretion of type I IFNs which facilitates radiotherapy, were significantly higher in responders compared with non-responding patients. Subsequent mouse knock-out studies uncovered that the gut microbiome–liver axis actively regulates host cytotoxic T cell responses through bacterial-derived c-di-AMP/GMP, which is posited to be acting as an agonist of the host cGAS/STING pathway. Antibiotic treatment significantly counteractes the anti-tumor effects by suppressing antigen presentation and inhibiting effector T cell responses. Elucidation of the mechanisms governing microbiota-mediated immune suppression will lead to strategies that could enhance the anti-tumor immune response.

Preclinical and clinical data from Shiao et al. (2021) revealed that the depletion of commensal bacteria is associated with overgrowth of commensal fungal populations and impaired IR-induced anti-tumor action primarily through CD8^+^ T cell exhaustion. Depletion of fungi leads to reduced tumor volume and improved survival of mice receiving IR compared with controls or mice receiving antifungals or IR alone. Sequencing studies of clinical samples from patients with triple-negative breast cancer revealed that intratumoral expression of Dectin-1, a fungal pathogen sensor, is inversely correlated with survival.

The critical role of metabolites secreted by the gut microbiome has been elucidated by recent studies; short-chain fatty acids have been found to mediate immune cell function and modulate responses to radiotherapy (Uribe-Herranz et al., 2020; Yang et al., 2021). Preclinical data indicate that depletion of vancomycin-sensitive bacteria enhances the anti-tumor activity of radiotherapy via eliminating immunosuppressive metabolites (butyrate and propionate). Vancomycin augments radiotherapy-induced abscopal effects by increasing DC antigen presentation and promoting tumor-associated antigen-specific CD8^+^ T cell priming (Uribe-Herranz et al., 2020). In another study, oral supplementation of vancomycin-sensitive bacteria (Lachnospiraceae) increased butyric acid levels in circulation and tumors, suppressed IR-induced type I IFN responses, and diminished the anti-tumor effects of IR (Yang et al., 2021).

Tumor microbiota effects on chemotherapy or immunotherapy outcomes have been reported. Gammaproteobacteria, which is abundant in human pancreatic ductal adenocarcinoma, expresses cytidine deaminase that metabolizes gemcitabine. Since gemcitabine is used as a radiosensitizer in some gastrointestinal malignancies, gammaproteobacteria is a source of treatment resistance (Geller and Straussman, 2017; Xuan et al., 2014). On the other hand, intratumoral translocation of *Bifidobacterium* has been found to promote the anti-tumor efficacy of anti-CD47 treatment (Shi et al., 2020).

The commensal microbiome also plays a significant role in shaping the side effects of radiotherapy. Investigating the specific mechanisms through which the microbiota influences these side effects is essential for optimal radiotherapy outcomes. Several studies (Atarashi et al., 2013; Nam et al., 2013; Wang et al., 2015) have demonstrated that patients who developed acute diarrhea after radiotherapy exhibit significant alterations in their gut microbiota composition compared with healthy volunteers or those without diarrhea. Gut microbial–derived propionate and tryptophan pathway metabolites (1H-indole-3-carboxaldehyde, kynurenic acid) provide long-term radioprotection in vivo, alleviating gastrointestinal syndromes and prolonging survival (Guo et al., 2020). The use of diets, fecal microbiota transplants, and the administration of specific microbial strains or microbial metabolites have been investigated for radiation-induced enteritis.

The multifaceted role of the microbiota in the context of radiotherapy or the combination of radiotherapy and ICB underlies a dynamic interplay between anti-tumor immunity, immune suppression, and treatment-associated side effects, and has potential importance for personalized medicine.

## New directions: Preclinical

### Radiation-induced production of growth factors

An interesting, understudied aspect of IR is its potential pro-tumorigenic/pro-metastatic effect. IR can enhance motility and invasiveness of cancer cells (Kawamoto et al., 2012; Wild-Bode et al., 2001; Zhang et al., 2011) and cause increased stemness and radioresistance of surviving clones, and has therefore been implicated in the facilitation of multiple steps of the metastatic cascade (Lee et al., 2017). Another important process of IR-facilitated metastasis is the induction of EMT (Gomez-Casal et al., 2013; Kawamoto et al., 2012; Mempel et al., 2024; Zhang et al., 2011). These events are enhanced by IR-induced production of growth factors, chemokines, and cytokines, which influence tumor development and metastasis (Hallahan et al., 1989; Veeraraghavan et al., 2011). IR has been described to induce the production of TGF-α (Kim et al., 1997), TGF-β (Park et al., 2001), TNFα (Dittmann et al., 2005; Rodemann et al., 2007), platelet-derived growth factor (Formenti et al., 2018) and VEGF (Hotta et al., 2021), and growth factor receptors such as EGFR (Kelly et al., 2008; Ready et al., 2010). Little is known about the effects of these molecules on anti-tumor immune response in the context of IR, especially in the setting of metastatic/systemic disease. Systemic TGF-β blockade is currently being examined in combination with focal irradiation in metastatic breast cancer (Wrona et al., 2021).

### New technologies: Nanoparticles

Nanoparticles, characterized by their unique physicochemical, magnetic, optical, or catalytic properties, serve as a sophisticated platform to minimize off-target effects and enhance the overall therapeutic efficacy of radiotherapy (Zhen et al., 2023). Among others, nanoscale metal-organic frameworks (MOF) can promote radioimmunotherapy efficacy (Lu et al., 2018), as well as the effects of radiotherapy in combination with chemotherapy and photodynamic therapy (He et al., 2016; Li et al., 2021b). A phase 1 dose-escalation study of RiMO-301, a hafnium-based MOF, has recently shown promising anti-tumor efficacy in combination with palliative radiotherapy (Koshy et al., 2023).

Self-assembling nanoscale coordination polymers (NCPs) (Liu et al., 2014), which take advantage of the enhanced permeability and retention effect and the versatility of nanoparticles, have successfully delivered chemotherapies (Duan et al., 2019; Liu et al., 2014, 2023) and also bacterial-derived STING agonists (Dosta et al., 2023; Wang-Bishop et al., 2023). Zinc c-di-AMP NCPs have shown effective tumor suppression by activation of endothelial STING and TAM reinvigoration, and they can bolster radio- and immunotherapy efficacy in immunologically cold and radioresistant tumor types (Yang et al., 2022).

### New technologies: Bispecific antibodies and fusion proteins

Bispecific antibodies—recombinant molecules containing two different antigen-identifying domains—offer a versatile approach to modulate immune responses in combination with radioimmunotherapy (Wei et al., 2022). Promising immune-activating effects have been achieved upon simultaneous targeting of T cell coreceptor CD3ε and PD-L1, which has been shown to rejuvenate anti-tumor T cell response by alleviating DC-mediated PD-L1 suppression while increasing B7-1&2 costimulation (Liu et al., 2018, 2021). DC-mediated anti-tumor activity has also been the basis of trials investigating bispecific antibodies that target CD40 activation in combination with CD11c, DEC-205, or CLEC9A (Salomon et al., 2022). Fusion proteins, which, similar to bispecific antibodies, contain functional domains from distinct molecules, offer another tailored strategy for immune modulation alongside radiotherapy (Xue et al., 2021). Recently, promising results have been achieved by combining an IL-2 mutein/Fc fusion protein with IL2 receptor β (Hsu et al., 2021) as well as a cetuximab-based IL-10 fusion protein for EGFR-targeted delivery of IL-10 to tumors (Qiao et al., 2019). Further promising new directions currently lie within the growing field of theragnostics, exemplified by promising fusion proteins such as Lu-177-DOTA octreotate for somatostatin receptor-expressing neuroendocrine tumors (Howe et al., 2020), or the recently FDA-approved Lu-177-Vipivotide-Tetraxetan for patients with metastatic castration-resistant prostate cancer (Fallah et al., 2023).

## Clinical studies

### Clinical evidence for radiotherapy and immunotherapy combinations

The preclinical data discussed above inspired efforts to combine radiotherapy and immunotherapy in the clinic. In particular, immune checkpoint inhibitors are the most commonly used immunotherapeutic agents in radiotherapy and immunotherapy combinations for solid tumors. As such, these combinations are the focus of this section of our review. Clinical trials have investigated radiotherapy and ICB combinations in a variety of contexts from early-stage malignancy to advanced, widely metastatic disease.

### Early-stage malignancy

A randomized phase 2 trial investigated SBRT with or without concurrent and adjuvant nivolumab in patients with stage IA-IIB NSCLC. The investigators identified a significant benefit to event-free survival of 77% versus 53% at 4 years in favor of combined SBRT and ICB (Chang et al., 2023). Three phase 3 trials are currently investigating combined SBRT with other anti-PD-(L)1 therapies (NCT03833154, NCT04214262, and NCT03924869). Results from a randomized phase 2 trial including stage I-IIIA NSCLC patients receiving neoadjuvant durvalumab with or without SBRT published by Formenti and colleagues demonstrated an improved rate of major pathologic response in 53% of patients receiving the combined treatment versus 7% of patients receiving durvalumab alone, though disease-free survival was not significantly improved (Altorki et al., 2021, 2023). These trials suggest a benefit to concurrent ICB in the early-stage setting, at least in NSCLC.

### Locally advanced malignancy

Standard-of-care treatment for locally advanced cancers with radiotherapy most commonly requires the use of conventional fractionation (1.8–2 Gy per fraction) and treatment of a larger field, often including tumor-draining lymph nodes, which are at risk for microscopic spread of disease. Large-scale randomized trials of radiotherapy and ICB in this setting have investigated both concurrent and adjuvant ICB, with adjuvant trials being more successful. Trials of chemoradiotherapy with or without ICB in rectal adenocarcinoma, head and neck cancer, and NSCLC were negative for their primary endpoints (Kemp, 2023; Lee et al., 2021; Machiels et al., 2022; Mell et al., 2022; Rahma et al., 2021; Tao et al., 2023). Trials in glioblastoma multiforme—which is not “locally advanced” but which is treated with conventional fractionation and concurrent chemotherapy—examined the addition of nivolumab to standard-of-care chemoradiotherapy and were negative for an overall survival (OS) benefit (Lim et al., 2022; Omuro et al., 2023). Meanwhile, there is conflicting evidence for concurrent ICB in cervical cancer. The CALLA trial of chemoradiotherapy with or without concurrent and adjuvant durvalumab was negative for a 2-year progression-free survival (PFS) benefit, while the KEYNOTE-A18 study, which examined the addition of concurrent and adjuvant pembrolizumab, was positive for the same endpoint (Lorusso et al., 2024; Monk et al., 2023).

Except in cervical cancer, the only phase 3 trials showing a survival benefit with combined conventionally fractionated radiotherapy and ICB in the locally advanced setting use adjuvant rather than concurrent ICB. The PACIFIC trial examined chemoradiotherapy with or without adjuvant durvalumab in patients with locally advanced unresectable NSCLC. There was a significant, durable benefit to PFS and OS with the addition of durvalumab, setting the current standard-of-care for unresectable locally advanced NSCLC (Antonia et al., 2017, 2018; Spigel et al., 2022). Similarly, CheckMate-577 investigated adjuvant nivolumab following chemoradiotherapy and surgery in patients who underwent a margin-negative resection but had residual pathologic disease. Adjuvant nivolumab significantly improved disease-free survival in this population (Kelly et al., 2021).

One cause for the failure of trials of concurrent ICB in the locally advanced setting could be the use of elective nodal irradiation in this population. Preclinical models have shown that targeting the tumor-draining lymphatics attenuates the response to ICB by reducing CD8^+^ T cell chemoattractant chemokine signaling and type I IFN signaling, and also reducing the expansion of antigen-experienced T cells and type I DCs in the draining nodes (Darragh et al., 2022a; Marciscano et al., 2018; Saddawi-Konefka et al., 2022). Translational data from a phase I/Ib clinical trial in patients with locally advanced laryngeal or oral cavity cancer treated with preoperative SBRT and durvalumab followed by surgical resection identified an increase in activated T cells with SBRT and ICB compared with those patients who did not receive the trial treatments (Darragh et al., 2022a, 2022b). Taken together, these findings strongly suggest that elective nodal irradiation results in both local and systemic immunosuppression.

The use of elective nodal irradiation may counteract the T cell–stimulating effects of ICB in the tumor-draining lymph nodes, drawing in T cells that are then ablated by consecutive daily treatments. While the omission of elective nodal irradiation is an ongoing area of research in head and neck cancer, with one phase 2 trial showing the feasibility of targeting gross disease alone (Sher et al., 2023), it requires further investigation given that preclinical and clinical studies have shown higher rates of regional recurrence with omission of elective nodal irradiation (Darragh et al., 2022a; Ma et al., 2022). Ultimately, the current data in head and neck, NSCLC, and esophageal cancer support the use of adjuvant ICB following conventionally fractionated radiotherapy or chemoradiotherapy.

### Metastatic disease

Trials of radiotherapy and ICB combinations in the metastatic setting vary by dosing regimen, number of sites treated, and timing of radiotherapy relative to ICB. We categorize radiotherapy dosing regimens on these trials into “ablative” and “sub-ablative” regimens, with ablative treatments achieving a biologically equivalent dose of >100 Gy (assuming an α/β ratio of 10). Many sub-ablative radiotherapy trials were initiated based on preclinical data showing that doses below 12 Gy stimulate an immune response without activation of Trex1 and that doses of 7.5 Gy per fraction maximized tumor control and immunity without stimulating immunosuppressive Tregs (Schaue et al., 2012; Vanpouille-Box et al., 2017). These trials delivered SBRT to 24–30 Gy in 3–5 fractions to one or several metastases per patient (Formenti et al., 2018; Kim et al., 2023; Mahmood et al., 2021; McBride et al., 2021; Monjazeb et al., 2021; Pakkala et al., 2020; Papadopoulos et al., 2020; Schoenfeld et al., 2022; Spaas et al., 2023; Theelen et al., 2019). An early trial in NSCLC suggested that combination therapy with radiotherapy and anti-CTLA-4 treatment induced a robust immune response (Formenti et al., 2018), although randomized trials investigating the addition of sub-ablative radiotherapy to ICB were negative for their respective endpoints, showing no clear benefit to the addition of radiotherapy (Kim et al., 2023; Mahmood et al., 2021; Monjazeb et al., 2021; Pakkala et al., 2020; Schoenfeld et al., 2022; Spaas et al., 2023; Theelen et al., 2019). Several trials also investigated the addition of low-dose radiotherapy of 0.5–1 Gy with every cycle of immunotherapy or every 2 weeks, without demonstrating a survival benefit (Herrera et al., 2022; Monjazeb et al., 2021; Schoenfeld et al., 2022). While there is evidence that combined sub-ablative SBRT and ICB increase markers for immune activation, there is no evidence currently that this translates into improved outcomes in the metastatic setting.

Many of these same trials treated a single site of metastasis, leaving other metastases untreated by radiation, again with the purpose of stimulating a robust systemic immune response compared with ICB alone (Chen et al., 2023a; Formenti et al., 2018; Kim et al., 2017; Kwon et al., 2014; Maity et al., 2021; Pakkala et al., 2020; Papadopoulos et al., 2020; Theelen et al., 2019; Welsh et al., 2019). Several of these trials randomized patients to ICB with or without radiotherapy and failed to show a benefit to the addition of radiotherapy (McBride et al., 2021; Pakkala et al., 2020; Theelen et al., 2019). As with sub-ablative dosing, there is currently no evidence to support the use of single-site radiotherapy—whether ablative or sub-ablative—to induce an immune response at distant sites in patients with polymetastatic disease.

Studies of ablative SBRT to multiple—though not necessarily all—metastases have demonstrated that this approach is safe and well-tolerated (Bestvina et al., 2022; Foster et al., 2021; Luke et al., 2018; Welsh et al., 2020). A randomized trial of pembrolizumab with or without radiotherapy (50 Gy in 4 fractions or 45 Gy in 15 fractions for SBRT-ineligible lesions) in metastatic NSCLC patients did not identify a benefit to the addition of SBRT in all patients, though those with low PD-L1 expression did see improvement in outcomes (Welsh et al., 2020). A pooled analysis of this trial and a trial of pembrolizumab with or without sub-ablative single-site radiotherapy for metastatic NSCLC patients identified a PFS and OS benefit with combined modality treatment (Theelen et al., 2019, 2021; Welsh et al., 2020). Given the lack of benefit seen with sub-ablative and single-site radiotherapy in the polymetastatic setting, ablative SBRT is preferred given that a higher dose confers a higher probability of control of the treated lesion.

The current clinical data for combinations of radiotherapy and ICB offer evidence of what works, what doesn’t, and what requires further investigation. In early-stage NSCLC, concurrent SBRT and ICB have shown promise, with multiple confirmatory phase 3 trials underway. When treating larger fields with IR, however, concurrent ICB appears to add no benefit in head and neck and lung cancer and may even be detrimental to the systemic immune response. Adjuvant ICB following (chemo)radiotherapy is preferred in the locally advanced setting for these disease sites. There may be other disease sites that, like cervical cancer, do benefit from concurrent ICB under certain conditions. For metastatic disease, the addition of sub-ablative SBRT and single-site treatment to ICB has not shown benefit over ICB alone in a randomized setting. While certain populations may benefit from combined SBRT and ICB, further work is needed to determine who benefits and how best to augment the effects of combined treatment. Biomarkers for response to radiotherapy and ICB may aid in identifying candidates for treatment, with tumor aneuploidy being one promising candidate (Alessi et al., 2023; Spurr et al., 2022). Finally, the goal of metastasis-directed therapy must be to achieve tumor kill with ablative radiotherapy to as many sites as is safe and feasible. This approach is under investigation in the SABR-COMET 10 and ARREST trials (Bauman et al., 2021; Palma et al., 2019).

Over a century of research findings, both basic and clinical, provide the foundations for the data described herein. If not for space limitations, a more comprehensive and inclusive review of the literature and important contributions to the field would have been included.
